# Shortening and validation of the Patient Engagement In Research Scale (PEIRS) for measuring meaningful patient and family caregiver engagement

**DOI:** 10.1111/hex.13227

**Published:** 2021-03-17

**Authors:** Clayon B. Hamilton, Alison M. Hoens, Annette M. McKinnon, Shanon McQuitty, Kelly English, Lisa D. Hawke, Linda C. Li

**Affiliations:** ^1^ Department of Physical Therapy University of British Columbia Vancouver BC Canada; ^2^ Arthritis Research Canada Richmond BC Canada; ^3^ Arthritis Patient Advisory Board Arthritis Research Canada Richmond BC Canada; ^4^ Centre for Addiction and Mental Health Toronto ON Canada; ^5^ University of Toronto Toronto ON Canada

**Keywords:** evaluation instruments, family caregiver, patient and public involvement, patient‐oriented research, psychometrics, reliability and validity

## Abstract

**Objective:**

To shorten the Patient Engagement In Research Scale (PEIRS) to its most essential items and evaluate its measurement properties for assessing the degree of patients’ and family caregivers’ meaningful engagement as partners in research projects.

**Methods:**

A prospective cross‐sectional web‐based survey in Canada and the USA, and also paper‐based in Canada. Participants were patients or family caregivers who had engaged in research projects within the last 3 years, were ≥17 years old, and communicated in English. Extensive psychometric analyses were conducted.

**Results:**

119 participants: 99 from Canada, 74 female, 51 aged 17‐35 years and 50 aged 36‐65 years, 60 had post‐secondary education, and 74 were Caucasian/white. The original 37‐item PEIRS was shortened to 22 items (PEIRS‐22), mainly because of low inter‐item correlations. PEIRS‐22 had a single dominant construct that accounted for 55% of explained variance. Analysis of PEIRS‐22 scores revealed the following: (1) acceptable floor and ceiling effects (<15%), (2) internal consistency (ordinal alpha = 0.96), (3) structural validity by fit to a Rasch measurement model, (4) construct validity by moderate correlations with the Public and Patient Engagement Evaluation Tool, (5) good test‐retest reliability (ICC_2,1_ = 0.86) and (6) interpretability demonstrated by significant differences among PEIRS‐22 scores across three levels of global meaningful engagement in research.

**Conclusions:**

The shortened PEIRS is valid and reliable for assessing the degree of meaningful patient and family caregiver engagement in research. It enables standardized assessment of engagement in research across various contexts.

**Patient or public contribution:**

A researcher‐initiated collaboration, patient partners contributed from study conception to manuscript write‐up.

## 1 INTRODUCTION

Increasingly, patients, family caregivers and the public actively engage with other stakeholders in health research projects in various contexts globally.^1^, ^2^, ^3^, ^4^ This engagement is often dynamic and hands‐on—for example, co‐developing documents, participating in decision‐making and providing advice on activities at any and all stages that shape the research process and outcomes.^5^ The extent to which they are actively involved in decision‐making creates a spectrum of engagement—at the lowest level they are simply informed about research; at the highest, they lead research activities and have primary decision‐making authority.^6^ Over the last decade, there has been a substantial increase in support for the unique and impactful contribution patients and family caregivers make to improve the relevance, appropriateness and use of research to serve the interests and needs of patients.^2^

It is expected, and even mandatory in some circumstances, to include patients and family caregivers in research teams as stakeholders with a personal interest in health research.^2^ The Canadian Institutes of Health Research, for example, outlines in its Strategy for Patient‐Oriented Research (SPOR) initiative that patients should be engaged in ‘active and meaningful collaboration’ as partners in the research process.^7^ There are currently numerous frameworks, models, best practices and other guidelines to support this goal^8^, ^9^, ^10^, ^11^, ^12^; but if we want to achieve sustainable improvements in how acceptable, feasible, rigorous and relevant research studies are in terms of patients’ realities, we need good quality patient engagement.^3^

While it is promoted, practiced and studied, there is little quantitative evidence on how patient engagement in research increases the quality of health research to improve health and health care.^13^ This could be due in part to a lack of validated measurement tools to determine the quality of patient engagement. A 2018 systematic review by Boivin and colleagues found that 27 existing patient and public evaluation tools, capturing both qualitative and quantitative data, needed more scientific rigour and patient engagement in their design and write‐ups.^14^ A 2020 scoping review on the evaluation of patient partnership in research revealed there were no quantitative assessments: all the identified studies used a qualitative approach.^13^ Quantitative assessments provide more objective and efficient ways of measuring the quality of engagement,^15^ thus enabling researchers to move from generating to testing hypotheses. By using validated quantitative measures of patient engagement in research, we can evaluate the effectiveness of engagement methods and strategies. This evaluation is vital to improve the quality of partnerships with patients in research projects and across research networks and research initiatives, and provide more generalizable findings that move beyond lessons learned and reflective narratives.

Shortly after Boivin et al’s 2018 systematic review was published,^14^ the Patient Engagement In Research Scale (PEIRS) was published as the first tool designed to measure the degree of meaningful patient engagement in research on project teams.^16^ The PEIRS is based on an empirical conceptual framework enhanced with a literature review,^5^ recognized as a promising and important tool for the evaluation of patient and family caregiver engagement in research.^13^, ^17^, ^18^, ^19^, ^20^ The framework outlines the key components of and defines meaningful patient engagement in research as the planned, supported and valued involvement of patients in the research process, which facilitates their contributions and offers a rewarding experience.^5^ While the original 37‐item PEIRS has undergone face and content validation, most of its measurement properties have not yet been assessed.^16^ Furthermore, informal conversations with researchers revealed the length of PEIRS might hinder its implementation. This study sought, therefore, to (1) reduce the respondent burden of the PEIRS by creating a shortened version containing its most essential elements, and (2) evaluate its measurement properties (internal consistency, structural and construct validity, reliability and interpretability) for assessing the degree of meaningful engagement of patients and family caregivers as partners in research projects.^16^

## 2 METHODS

### 2.1 Study design

This survey used two sampling strategies: a web‐based survey and a paper‐based survey through collaboration with LDH. Eligible individuals were patients or family caregivers who had engaged as partners in research within the last three years, were 18 years or older (≥17 years for the paper survey) and could communicate in English. The University of British Columbia Behavioural Research Ethics Board approved this study (REB#H15‐00217).

### 2.2 Web‐based survey

The web‐based survey recruitment, using the Qualtrics survey tool (https://ubc.qualtrics.com), started in Canada in October 2018, extended to the United States in October 2019 and ended in both countries in March 2020. It entailed a multimodal approach involving internet‐mediated and traditional methods with a study recruitment poster.^21^ An email invitation containing the recruitment poster was sent first to participants from previous studies by the lead author (CBH) and then to selected patient partners, researchers and relevant organizations. The poster was also posted on websites, in newsletters and on social media platforms (including Twitter and Facebook) via the accounts of the research team members, research organizations and networks, community organizations, and research‐affiliated patient groups and organizations in Canada and the United States. In addition, we emailed researchers who engaged with patient partners and had (1) cited either of two frameworks on patient engagement by the lead author (CBH) and team,^5^, ^22^ (2) published in a special 2019 edition of a Canadian Medical Journal or (3) presented at certain webinars. Finally, participants were asked to share the opportunity with other potential participants.

Potential participants completed an eligibility screening form. Eligible individuals were emailed a personal survey link and asked to complete the questionnaires within three weeks. Eligible individuals were sent up to two reminders before the deadline and were contacted within a month of the deadline if they did not complete the survey. The web‐based survey comprised an informed consent page, demographic questions, the 37‐item PEIRS,^16^ an item of global meaningful patient engagement in research and the Public and Patient Engagement Evaluation Tool (PPEET) Participant Questionnaire.^17^ At the end of the survey, participants were asked to indicate their willingness to complete a second survey within two to seven days for test‐retest reliability. Demographic information was collected on age, gender identity, education level, ethnicity/racial identity, household income, province/state of residence, type of patient partner, phase of research, jurisdictional scope of research team and description of the project being reported on. Participants had a 1 in 20 chance of receiving a CAD$75 (or US$50) gift card for participating.

### 2.3 Paper‐based survey

The paper‐based survey involved youths who had lived experience of substance use, had attended one of two 1‐day youth summit events on opioid interventions and services geared towards substance misuse for at‐risk youths and new users in Ontario, Canada, and met similar criteria to those specified in the web‐based survey. The summits were co‐designed and co‐facilitated by youth and project allies, supported by a ‘developmentally informed’ youth engagement strategy and not by the PEIRS’s conceptual framework.^23^ At the end of the summit, the youths who participated were asked to complete the same questions used in the web‐based survey.

### 2.4 Patient Engagement In Research Scale (PEIRS)

The PEIRS is a self‐administered 37‐item questionnaire completed by patient partners (including family caregiver partners) to determine their degree of meaningful engagement in research as an indicator of the quality of their engagement in a research project.^16^ Each item requires respondents to reflect on their experiences as a research partner in a specific project. PEIRS captures key elements of eight themes from a conceptual framework for meaningful engagement in research.^5^, ^16^ These themes align with the seven sections/subscales of the PEIRS: procedural requirements (PR, 14 items), convenience (CN, 4 items), contributions (CT, 4 items), two themes combined as ‘team environment and interaction’ (T, 5 items), support (SU, 3 items), feel valued (FV, 3 items) and benefits (BE, 4 items). Each item uses a 5‐point Likert scale (‘strongly agree’ to ‘strongly disagree’) we scored 4 to 0. It achieved good content and face validation.^16^

### 2.5 Global meaningful patient engagement

A single item that reads ‘Overall, how meaningful was your experience being a part of the research project?’ was used to capture participants’ perception of their global meaningful engagement in a research project. The item used a 5‐point response scale (5—extremely, 4—very, 3—moderately, 2—slightly and 1—not meaningful) co‐designed by our research team, including patient partners, for this project. Responses were reported with ‘not’ to ‘moderately’ meaningful grouped as a single category.

### 2.6 Public and Patient Engagement Evaluation Tool (PPEET)

The PPEET, first published in 2015 and updated in 2018, consists of three questionnaires (participant, project and organization questionnaires), each developed to assess the processes, outputs, and perceived impacts of engagement activities in health system organizations.^17^, ^24^ The participant questionnaire has two versions: one designed for one‐time engagement and the other for ongoing/long‐term engagement activities. As a seminal questionnaire widely used in Canada for evaluating patient engagement in research, we chose the PPEET for convergent validation to assess construct validity of the shortened PEIRS. We used the one‐time engagement version because its phrasing in past tense, as compared to present tense, aligned better with the PEIRS, which was developed for both one‐time and ongoing engagement activities. The PPEET has two demographic items, plus 19 experience items (including six open‐ended items), divided into four groups.^17^ We used 10 closed‐ended items that seemed relevant to engagement in research: PP1 to PP3 (items 3 to 5) for ‘communication and support of participation’, PP4 to PP7 (items 7 to 10) for ‘sharing your views and perspectives’, PP8 (item 12) for ‘impacts and influence of engagement initiative’, and PP9 and PP10 (items 17 and 18) for ‘final thoughts’. Item 16 was excluded because it needed tailoring for each respondent. We worded the items for participants’ views on a research ‘engagement initiative’. Each item used a 5‐point Likert scale, ranging from 1 for ‘strongly disagree’ to 5 for ‘strongly agree’.^17^, ^24^ It has undergone face and content validation but has not had its measurement properties evaluated. No scoring instructions were published for the PPEET.

### 2.7 Sample size considerations

Guided by the quality criteria from Terwee et al (2007) for the measurement properties of health status questionnaires,^25^ the target sample size was at least 100 participants for satisfactory evaluation of internal consistency and 50 participants for test‐retest reliability over two to seven days. Within this period, our research team which includes patient partners anticipated the respondent's engagement experiences would not change and previous responses forgotten.^25^ We aimed for seven participants per item for exploratory factor analysis.^25^

### 2.8 Patient engagement in the current study

This researcher‐initiated study was part of an ongoing three‐phase research project spanning more than 3 years of collaboration among researchers and four experienced patient partners as research team members. In the previous two phases, the patient partners co‐designed the conceptual framework and the PEIRS.^5^, ^16^ They were middle‐aged Caucasian women with arthritis diagnoses who self‐selected their engagement in this research team from an institutional patient advisory board (Arthritis Research Canada's Arthritis Patient Advisory Board). On the advisory board, they engage in an array of research‐related activities (https://www.arthritisresearch.ca/our‐team/arthritis‐patient‐advisory‐board/). The patient partners have engaged in all phases of the current study,^22^ contributing to study protocol, recruiting participants by sharing information actively and passively through their networks, and discussing the objectives as well as expected and proposed findings of this study through in‐person and virtual/teleconference meetings. They assisted in writing this paper by reviewing an early draft and providing feedback. Patient partners were offered an honorarium to acknowledge their contributions consistent with current Canadian guidance.^26^

### 2.9 Data analysis

We calculated descriptive and inferential statistics to evaluate and refine the measurement properties of the PEIRS. Most statistical analyses were performed using R version 3.6.1 (The R Foundation, Vienna, Austria) in RStudio version 1.3.959, open‐sourced under an AGLP v3 licence, with a few R packages, including blandr, ggpubr, irr, paran, and psych. Rasch analysis was performed using RUMM2030 (RUMM Laboratory Pty Ltd).

### 2.10 Internal consistency

This depicted how unified items were for measuring meaningful engagement in research.^27^ Because data collected using Likert scales are ordinal‐level (or categorical) data, internal consistency was evaluated using a polychoric correlation matrix of items. A resulting average inter‐item correlation between 0.20 and 0.40 is ideal; a lower value means items capture different constructs and higher values mean they capture narrowing ranges of the construct.^28^ The corrected item‐test correlation used a polyserial correlation coefficient, with a criterion of ≥0.4 for retaining items.^29^ The ordinal coefficient alpha (criterion: ≥0.70), which is conceptually equivalent to Cronbach's alpha, was calculated using a polychoric correlation matrix.^30^ Cronbach's alpha with the same criterion was calculated as a typically reported coefficient. We inspected the inter‐item correlation matrix of the PEIRS and removed items that were too lowly (<0.30) or highly (>0.80) correlated.^31^ The reduction step was conducted through an iterative process for refinement of the PEIRS and was informed by internal consistency analysis, the distributions of item responses and team discussions. The expected outcome was a parsimonious set of items that are internally consistent and have minimal respondent burden.

### 2.11 Structural validity

Once the PEIRS had been refined for adequate internal consistency, we assessed its underlying construct.^27^ When a participant had <15% of missing responses for the PEIRS, the item‐level mean rounded to the nearest whole number was imputed.^32^ A Kaiser‐Meyer‐Olkin measure for sampling adequacy of 0.93 confirmed a sufficient sample size and data to proceed with factor analysis.^33^ Horn's parallel analysis, a more accurate test than eigenvalues‐greater‐than‐one rule and scree plot approach, indicated we could extract one factor from the PEIRS data.^34^ Exploratory factor analysis with principle axis factoring was then used to determine the items to retain in the factor and thus the questionnaire.^35^ Each item retained met an a priori factor loading criterion of ≥0.32.^35^

Rasch analysis uses probability estimates to inform evaluation and refinement of questionnaires.^36^ Adequate fit to the Rasch measurement model can lead to obtaining interval‐level scoring for questionnaires, which is desired for questionnaires’ use in comparative effectiveness research.^37^ A Rasch analysis was conducted on the retained items for fit to the partial credit version of the polytomous Rasch measurement model,^37^, ^38^ as it allows for variation between differences among item thresholds. We used Tennant and Conaghan's criteria for evaluating data fit to the Rasch model based on several fit statistics.^39^ Overall fit was investigated with three summary statistics: the item‐trait interaction chi‐square p‐value, mean person‐fit residual value and item‐fit residual value. A non‐significant (α > 0.05) chi‐square statistic would indicate a fit between the expected and observed structure of the PEIRS data. Person‐fit and item‐fit were achieved if (1) standardized (Z‐score) fit residuals were within ± 2.5 units, (2) the mean residual values approximated 0, and (3) the standard deviation of the mean residual values approximated 1. Additionally, we inspected category threshold graphs to evaluate if participants appropriately used the response categories of each item or if the items required rescoring. Items should have local independence, generally demonstrated by fit residual correlated below 0.3 between items.^39^ A measure has unidimensional properties when <5% of participants (estimated by the lower bound of the binomial 95% CI) has a significant t test between groups of negative and positively loading items based on their fit residuals.

As part of the Rasch analysis, we calculated the person separation index. The person separation index is interpreted similarly to Cronbach's alpha, and values >0.85 would mean the PEIRS is appropriate for the assessment of individual patient partners.^39^ Finally, we assessed for differential item functioning (or item bias) to determine whether scores for any item differed by demographic characteristics (age, gender, education and income) when participants had similar overall PEIRS scores.^39^, ^40^

### 2.12 Construct validity

There are no reference standards for measuring meaningful patient engagement in research. Polyserial correlation coefficients were used to assess correlations between the refined PEIRS total scores and scores from each of the 10 items of PPEET. We hypothesized a moderate correlation of ~0.5 for each pair.^41^ We explored, using hypotheses of no significant difference (α < 0.05), the relationship between PEIRS scores and demographic variables (gender, age, education attainment, household income, ethnicity/racial groups) using a non‐parametric equivalent to one‐way analysis of variance (ANOVA) to determine whether the degree of meaningful patient engagement differed by groups.

### 2.13 Reliability and measurement error

We evaluated the extent to which repeated administration of PEIRS by participants with stable experiences provided similar PEIRS scores. Test‐retest reliability was calculated using an intraclass correlation two‐way random effects model (ICC_2,1_) with 95% confidence intervals (CIs). A value between 0.75 and 0.90 was interpreted as good reliability and above 0.90 deemed excellent.^42^, ^43^ The 95% limits of agreement (LOA) repeatability coefficient provided the bounds of random differences between PEIRS scores that 95% of participants would expect to have after repeated administration of the PEIRS.^44^ We calculated the standard error of measurement (SEM) and the minimal detectable change at a 90% confidence level (MDC_90_) with 95 CIs.^25^, ^45^

### 2.14 Interpretability

We explored the extent to which qualitative meaning can be assigned to PEIRS scores.^25^ We tested the hypothesis that higher (more favourable) PEIRS scores will be associated with higher (more favourable) self‐reported levels of engagement as a research partner. The latter was indicated by the global meaningful engagement measure. When the polyserial correlation coefficient was >0.40 between PEIRS and the global engagment measure scores, we analysed three levels of global meaningful engagement (‘no to moderate’, ‘very’, and ‘extreme’) as this allowed for an adequate number of respondents per level. We had estimated a sample size requirement of 52 participants per level for a moderate effect size.^46^ When the assumptions of normality were not met, we used the Kruskal‐Wallis test to identify any statistically significant difference in PEIRS scores among three levels of meaningful engagement. When the results were statistically significant, we performed a post hoc pairwise Mann‐Whitney test with p‐values controlled for the three comparisons using Bonferroni adjustment. Effect size using results from the Mann‐Whitney test results was calculated as Cohen's *d* = 2r/√(1−r^2^), where *r* = *z*/√n, *z* was the *z*‐score value obtained from the Mann‐Whitney test and n was the total sample used per comparison.^47^ We considered ≥0.41 to <1.15 as small and the minimally relevant effect size, ≥1.15 to <2.70 as moderate, and ≥2.70 as large effects.^48^

## 3 RESULTS

### 3.1 Survey cohort

Table 1 summarizes the sample characteristics. For the web‐based survey, 119 individuals were screened, 106 were eligible, and 84 completed it. In total, 119 participants completed both versions of the survey; 99 (83.2%), including 35 from the youth summits, were from Canada. The majority were female (62.2%) or aged about equally between 17 to 35 years (42.9%) and 36‐65 years (42.0%). Most participants had completed some post‐secondary education up to a bachelor's degree (50.4%), and most had a household income of between US$24 000 and US$80 000. The majority (91.5%) identified as patient partners, with some of those also identifying as a family/friend/unpaid caregiver partner. The largest portion (43.7%) of participants were involved in local research teams, and participants’ research projects were predominantly (71.4%) in the carrying‐out phase.

**TABLE 1:** Characteristics of the study sample and corresponding PEIRS‐22 scores

Characteristics	Study sample Number (%), n = 119	PEIRS‐22
		Mean (standard deviation), n = 117
Country		
Canada	99^a^ (83.2)	84.34 (15.7)
United States	20 (16.8)	85.75 (13.6)
Gender		
Male	32 (26.9)	81.38 (15.4)
Female	74^a^ (62.2)	86.43 (13.4)
Gender‐diverse	10 (8.4)	82.9 (27.0)
Other/Missing	3 (2.5)	80.0 (4.0)
Age in years		
17‐35	51^b^ (42.9)	86.4(13.0)
36‐65	50^b^ (42.0)	83.4 (18.6)
≥65	17 (14.3)	83.2 (11.6)
Prefer not to say	1 (0.8)	–
Education		
High school or less	28 (23.5)	87.14 (12.7)
Some post‐secondary to bachelor's degree	60^a^ (50.4)	84.9 (17.9)
Master's, doctoral degree or above	30 (25.2)	81.67 (12.0)
Other	1 (0.8)	–
Household income (US dollars)		
Under $24 000	30^b^ (25.4)	85.31 (13.9)
$24 001 to $80 000	34^b^ (28.8)	81.94 (18.2)
Over $80 000	28 (23.7)	83.39 (17.3)
Prefer not to say	26 (22.0)	87.92 (10.1)
Ethnicity/Racial group^c^		
Indigenous	16 (13.4)	91.0 (9.6)
White	74^b^ (62.2)	83.9 (16.3)
Other	29^b^ (24.4)	83.6 (15.1)
Type of partner^d^		
Patient	109 (91.5)	84.3 (15.8)
Family/Friend/Unpaid caregiver	22 (18.5)	87.4 (9.8)
Type of team^e^		
Local	52^a^ (43.7)	86.4 (10.2)
Regional	15 (12.6)	87.0 (11.3)
National	41 (34.5)	81.1 (19.7)
International	8 (6.7)	85.6 (15.1)
Research stage^f^		
Preparation	33 (27.7)	84.1 (12.5)
Carrying‐out	85^b^ (71.4)	84.8 (16.3)
Dissemination	37 (31.1)	85.6 (10.9)

^a^ Indicates 2 participants missing in data analysis.

^b^ Indicates 1 participant missing in data analysis.

^c^ The Indigenous category includes people of mixed background. The ‘other’ category includes people of other mixed backgrounds, and those who identified as being of African descent (Black, n = 11), Asian, Chinese, West Asian, Filipino, Hebrew, Jewish, Latin American or Portuguese.

^d^ At‐risk youths would prefer being identified as person with lived experience rather than as patients.

^e^ Local means within an organization. Regional means spread across a single province/territory in Canada or state/metropolitan area in the USA. National means spread across two or more provinces/territories or state/metropolitan area. International means Canada/USA and one or more other countries.

^f^ Preparation includes identify/prioritize research question and seek funding. Carrying‐out includes recruit participants, collect data, analyse data and interpret data. Dissemination includes share results, help findings to be used and by extension assess research impact.

### 3.2 Descriptive statistics of survey

All 37 items of the PEIRS had response options of ‘strongly agree’, and 28 included response options of ‘strongly disagree’ to cover the ends of the response categories (see Table 2). One item (PR1) had ‘neutral’ as its least favourable response, and eight items (PR3 to PR8, PR11 and BE3) had ‘disagree’. The mean for each item was above ‘agree’, and the median was either ‘agree’ or ‘strongly agree’. While not important for the total scores, the item‐level ceiling effect varied between 41.2% and 71.3%, and the floor effect varied between 0.8% and 3.4%. A total of 15.9% of participants had missing items for the PEIRS. Seven items had no missing data. Missing data varied from 12 participants with one missing item and two participants with four missing items. Two participants who completed the paper‐based survey were removed for missing 6 and 23 items on PEIRS, respectively. The final study sample was 117 participants when evaluating the PEIRS. The refined version of the PEIRS discussed in this paper displayed no substantial floor effect (14.5%) or ceiling effect (0%) for its total scores. The refined PEIRS scores were calculated for a possible range of 0 (no degree of meaningful engagement) to 100 (high degree of meaningful engagement). The total scores ranged from 11 to 100, with a mean of 84.6 (SD = 5.35), for the initial testing, and from 52 to 100, with a mean of 83.3 (SD = 13.1), for retesting.

**TABLE 2:** Descriptive statistics for each item of the PEIRS (N = 119)

PEIRS Item^a^	Missing	Mean	SD	Median	Max	Min	Floor effect	Ceiling effect	Correlation with PEIRS‐22^b^ (N = 117)
PR1	0	3.7	0.51	4	4	2	1	71.3	0.30
**PR2**	**1**	**3.4**	**0.81**	**3**	**4**	**0**	**1.7**	**50.4**	**0.79**
PR3	2	3.2	0.85	4	4	1	1	42.9	0.68
PR4	2	3.5	0.67	4	4	1	1	61.3	0.51
PR5	0	3.6	0.56	4	4	1	1	68.1	0.57
PR6	0	3.4	0.72	4	4	1	1	51.3	0.47
PR7	0	3.4	0.81	4	4	1	1	54.6	0.86
PR8	2	3.5	0.75	4	4	1	1	60.5	0.50
**PR9**	**1**	**3.4**	**0.78**	**4**	**4**	**0**	**1.7**	**56.3**	**0.97**
**PR10**	**0**	**3.3**	**0.82**	**3**	**4**	**0**	**1.7**	**48.7**	**0.73**
**PR11**	**0**	**3.2**	**0.89**	**3**	**4**	**0**	**1.7**	**43.7**	**0.76**
**PR12**	**1**	**3.3**	**0.87**	**4**	**4**	**0**	**1.7**	**52.1**	**0.91**
**PR13**	**0**	**3.2**	**0.95**	**3**	**4**	**0**	**2.5**	**46.2**	**0.94**
**PR14**	**1**	**3.6**	**0.73**	**4**	**4**	**0**	**0.8**	**66.4**	**0.95**
**CN1**	**3**	**3.1**	**0.96**	**3**	**4**	**0**	**1.7**	**41.2**	**0.83**
CN2	2	3.2	0.99	3	4	0	2.5	45.4	0.65
**CN3**	**3**	**3.4**	**0.81**	**4**	**4**	**0**	**2.5**	**51.3**	**0.87**
**CN4**	**1**	**3.6**	**0.74**	**4**	**4**	**0**	**1.7**	**68.1**	**1.0**
**CT1**	**4**	**3.6**	**0.67**	**4**	**4**	**0**	**0.8**	**63.9**	**1.0**
**CT2**	**1**	**3.5**	**0.73**	**4**	**4**	**0**	**0.8**	**63.9**	**0.99**
CT3	2	3.6	0.67	4	4	0	0.8	64.7	0.99
**CT4**	**1**	**3.4**	**0.73**	**4**	**4**	**0**	**0.8**	**54.6**	**0.76**
T1	4	3.5	0.85	4	4	0	2.5	63.0	1.0
**T2**	**1**	**3.2**	**1.01**	**3**	**4**	**0**	**3.4**	**45.4**	**0.86**
T3	1	3.5	0.77	4	4	0	1.7	63.0	1.0
T4	3	3.5	0.83	4	4	0	1.7	63.0	1.0
**T5**	**1**	**3.5**	**0.79**	**4**	**4**	**0**	**1.7**	**61.3**	**0.95**
**SU1**	**2**	**3.3**	**0.87**	**4**	**4**	**0**	**1.7**	**49.6**	**0.82**
**SU2**	**3**	**3.4**	**0.85**	**4**	**4**	**0**	**0.8**	**52.9**	**0.96**
SU3	1	3.1	0.99	3	4	0	0.8	45.4	0.39
**FV1**	**2**	**3.5**	**0.81**	**4**	**4**	**0**	**1.7**	**60.5**	**0.99**
FV2	1	3.6	0.76	4	4	0	1.7	67.2	1.0
**FV3**	**2**	**3.2**	**1.02**	**3.5**	**4**	**0**	**3.4**	**48.7**	**0.78**
**BE1**	**2**	**3.6**	**0.68**	**4**	**4**	**0**	**1.8**	**68.9**	**0.95**
**BE2**	**3**	**3.2**	**0.85**	**3**	**4**	**0**	**1.8**	**44.5**	**0.82**
BE3	3	3.5	0.70	4	4	1	0	53.8	0.58
**BE4**	**3**	**3.4**	**0.81**	**4**	**4**	**0**	**0.8**	**52.9**	**0.83**

Note

Rows with bold texts indicate the items retained in the PEIRS‐22.

^a^ The codes correspond to the items in the 37‐item PEIRS: procedural requirements (PR, 14 items), convenience (CN, 4 items), contributions (CT, 4 items), two themes combined as ‘team environment and interaction’ (T, 5 items), support (SU, 3 items), feel valued (FV, 3 items) and benefits (BE, 4 items). Each item uses a 5‐point Likert scale scored 4 (‘strongly agree’) to 0 (‘strongly disagree’).

^b^ Item responses missing for each participant were replaced with mean of the items completed by that participant. The correlation coefficient was not corrected for overlap between item and scale.

### 3.3 Item reduction

The inter‐item correlation matrix of the 37‐item PEIRS revealed multiple negative or low (<0.10) correlations of eight items (PR1, PR3 to PR8 and BE3) with other items. These eight items also had the most limited coverage of their response categories. They were removed for weak fit with the other items to improve internal consistency for a PEIRS without subscales (see Appendix A for removed items). SU3 was removed for low correlations (<0.30) with several of the remaining items; T4 was removed for too high a correlation (>0.80) with T5, T1 for high correlations (≥0.78) with T3 and T5, and FV2 for high correlations (≥0.74) with T4 and FV1. The difference between T4 and T5 was affirming ‘mutual respect’ versus affirming ‘trust’, and either one could have been retained. Subsequently, a preliminary run of Rasch analysis with the remaining 24 items of PEIRS informed removal of CN2 for misfit with the other items (fit residual = 3.24) and removal of CT3 for strong local dependency with CT1 (residual correlation ≥0.40). In total, 15 items were removed, resulting in a 22‐item version of the PEIRS (PEIRS‐22) (see Appendix B). The measurement properties reported in this study pertain to the PEIRS‐22, which retains items from each of the eight themes of the conceptual framework and at least two items from each of the seven sections of the original 37‐item PEIRS.^16^, ^22^

### 3.4 Measurement properties

#### 3.4.1 Internal consistency

The 22‐item PEIRS (PEIRS‐22) had no inter‐item correlation below 0.2 or above 0.8, with an average inter‐item correlation of 0.55. The corrected item‐total correlation varied between 0.65 (item CT4) and 0.78 (items CT1, CT2, SU2 and FV1). The ordinal alpha was 0.96 and Cronbach's alpha 0.96.

#### 3.4.2 Structural validity

With 5.3 participants per item, Horn's parallel analysis revealed one extractable factor. Subsequently, exploratory factor analysis revealed that all 22 items loaded onto the factor with factor loadings between 0.66 (item CT4) and 0.80 (item FV1) and explained 55% of the variance in the data. All but four items had factor loading above 0.70. Corresponding fit indices tested and accepted the hypothesis that one factor was sufficient for the data, as demonstrated by standardized root mean square residual = 0.5, Tucker Lewis index = 0.93 and root mean square error of approximation = 0.06 (90% CI = 0.05 and 0.08).

**FIGURE 1:**
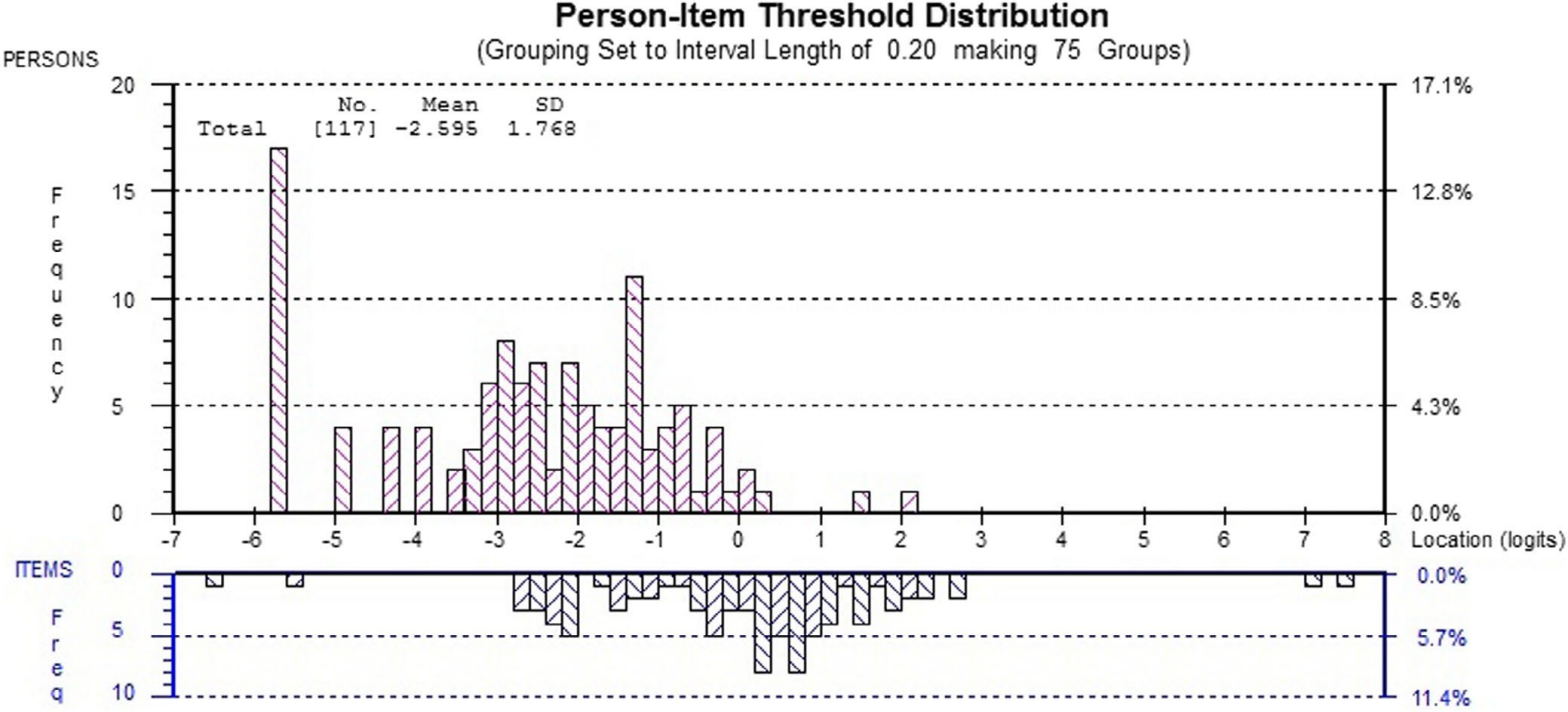
Person‐item‐fit threshold distribution. The participants’ fit distribution is shown in the upper histogram, and the items’ fit distribution in the lower histogram. The *x*‐axis represents the degree of meaningful patient engagement, with a higher number indicating a lower degree of engagement

**FIGURE 2:**
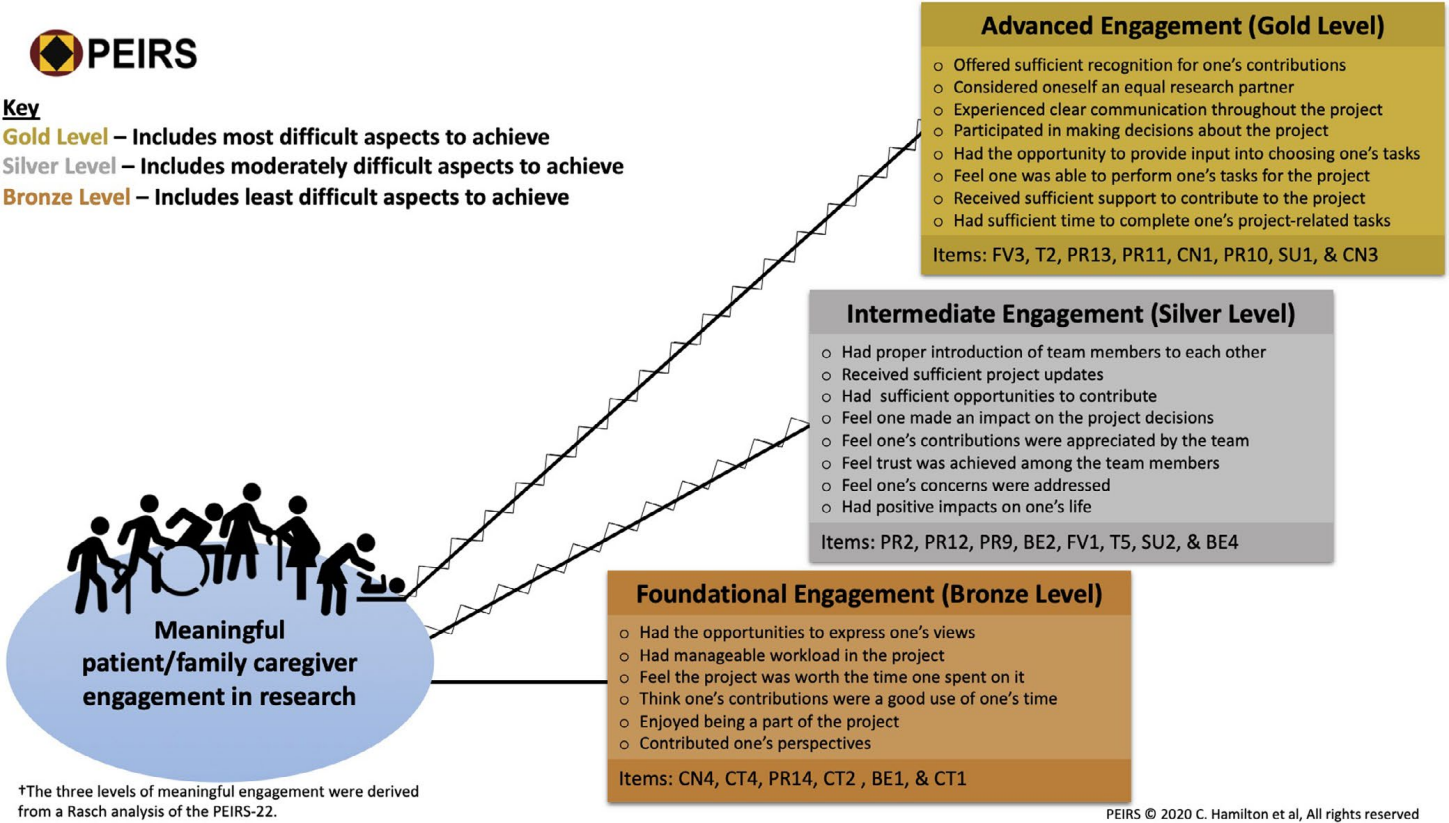
The journey along meaningful engagement in research. The figure depicts diverse patient/family caregiver partners’ journey in meaningful patient engagement in research projects. As they experience certain positive aspects of engagement, they move from foundational to advanced engagement

Rasch analysis showed an overall fit of PEIRS‐22 data to the Rasch measurement model α = 0.17, indicating the other fit statistics of Rasch analysis are reliable. The Rasch analysis had an excellent power of analysis from using 100 participants, after certain analyses required the removal of 17 (14.5%) participants with extreme Rasch‐generated scores. The summary fit statistics approached ideal criteria with mean residuals of 0.10 (SD = 1.09) for items and −0.26 (SD = 1.54) for persons. The person separation index was 0.89 when extreme person scores were included. Some slight local dependency (>0.3 and <0.4) of PR12 with PR13, PR12 with CN1, CN3 with SU1, and CN4 with CT1 remained in PEIRS‐22. Furthermore, all but PR13, CT1, BE1, BE2 and BE3 had disordered thresholds. This indicated that item response categories may benefit from combining scores from categories when calculating summative scores in order to produce an interval‐level scale. However, t tests were significant for 3.0% of participants, indicating the PEIRS‐22 could be interpreted as unidimensional for measuring meaningful patient engagement in research. We decided not to make further amendments to the PEIRS because, as depicted in Figure 1, the sample was not adequately targeted for participants with lower degrees of meaningful engagement. Rasch analysis also provided information on the ordering of items based on whether they tended to be endorsed for lower or higher degrees of the meaningful engagement in research construct captured by the 22‐item PEIRS. Figure 2 provides a practical illustration of this finding, in which we show the journey of patient/family caregiver partners from experiencing the foundational elements of meaningful engagement through to experiencing the advanced elements of meaningful engagement. We found that items CT1—‘I contributed by providing my perspective’, CT2—‘My contributions were a good use of my time’ and BE1—‘I enjoyed being a part of the project’ capture a low degree of meaningful engagement. Conversely, PR13—‘Communication within the research team was clear …’, T2—‘I was an equal partner …’ and FV3—‘I was offered sufficient recognition …’ capture high degrees of meaningful engagement. PR9—‘I had sufficient opportunities to contribute …’ is positioned about midway on the construct measured by PEIRS‐22.

#### 3.4.3 Test‐retest reliability and measurement error

**FIGURE 3:**
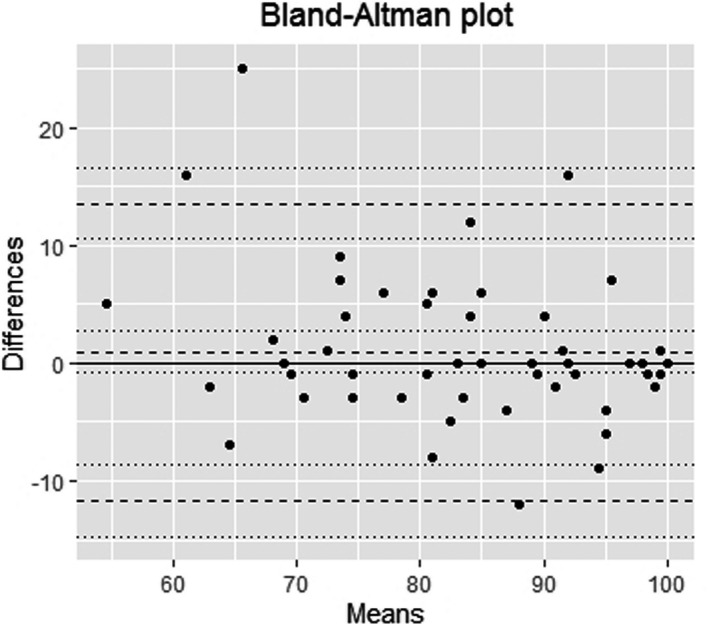
Bland‐Altman plot (n = 53). The plot shows the differences between the PEIRS‐22 score means of the two testing occasions are concentrated close to the zero line. Less than 5% of the differences were outside the limits of agreement zone (±2 standard deviation), and most differences are fewer than 10 units of the PEIRS‐22 scores. The average mean PEIRS‐22 scores vary between about 55 and 100, which indicates that participants’ responses concentrate at the higher end of the scale

Fifty‐three of 72 potential participants completed the PEIRS twice—a 73.6% response rate. Most (n = 49) took between 2 and 13 days to complete it, one completed it twice on the same day, and three took between 22 and 56 days to complete it. The ICC_2,1_ was 0.86 (95% confidence intervals = 0.77 and 0.92). Visual inspection of the Bland‐Altman plot (Figure 3) suggested overall small measurement error. The 95% limit of agreement was 13.56 (95% CI = 10.50 and 16.61) for the upper limit and −11.71 (95% CI = −14.71 and −8.65) for the lower. The SEM was 4.56 (95% CI = 3.61 and 5.51), and the MDC_90_ was 10.57 (95% CI = 8.37 and 12.77).

#### 3.4.4 Construct validity

Table 3 shows the polyserial correlations between the total scores of PEIRS‐22; each of the 10 items of the PPEET revealed six with moderate correlation coefficient values between 0.40 and 0.70. The PPEET item with the highest correlation was from the group of items on ‘final thoughts’, while the lowest correlation was from the group of items on ‘sharing one's views and perspectives’. Using the PEIRS‐22, the Kruskal‐Wallis test revealed no statistically significant differences for the median PEIRS scores among the categories of age, gender, education, income or race.

**TABLE 3:** Polyserial correlations of PEIRS‐22 scores with 10 individual items of PPEET‐Participant Questionnaire

PPEET‐Participant Questionnaire items^a^	Polyserial correlation with PEIRS‐22 scores
(PP1) Item 3—Clearly understand purpose	0.27
(PP2) Item 4—Supports needed were available	0.44^b^
(PP3) Item 5—Enough information to contribute	0.29
(PP4) Item 7—Express views freely	0.44^b^
(PP5) Item 8—Feel views were heard	0.58^b^
(PP6) Item 9—Shared views	0.50^b^
(PP7) Item 10—Broad range of perspective represented	0.26
(PP8) Item 12—Achieved objectives	0.56^b^
(PP9) Item 17—Overall satisfaction	0.59^b^
(PP10) Item 18—Good use of time	0.67^b^

^a^ The item numbers refer to the item in the Public and Patient Engagement Evaluation Tool (PPEET)‐Participant Questionnaire for one‐time engagement, and the codes correspond to those used specifically for the current study.

^b^ Moderate correlation coefficient value.

#### 3.4.5 Interpretability

**FIGURE 4:**
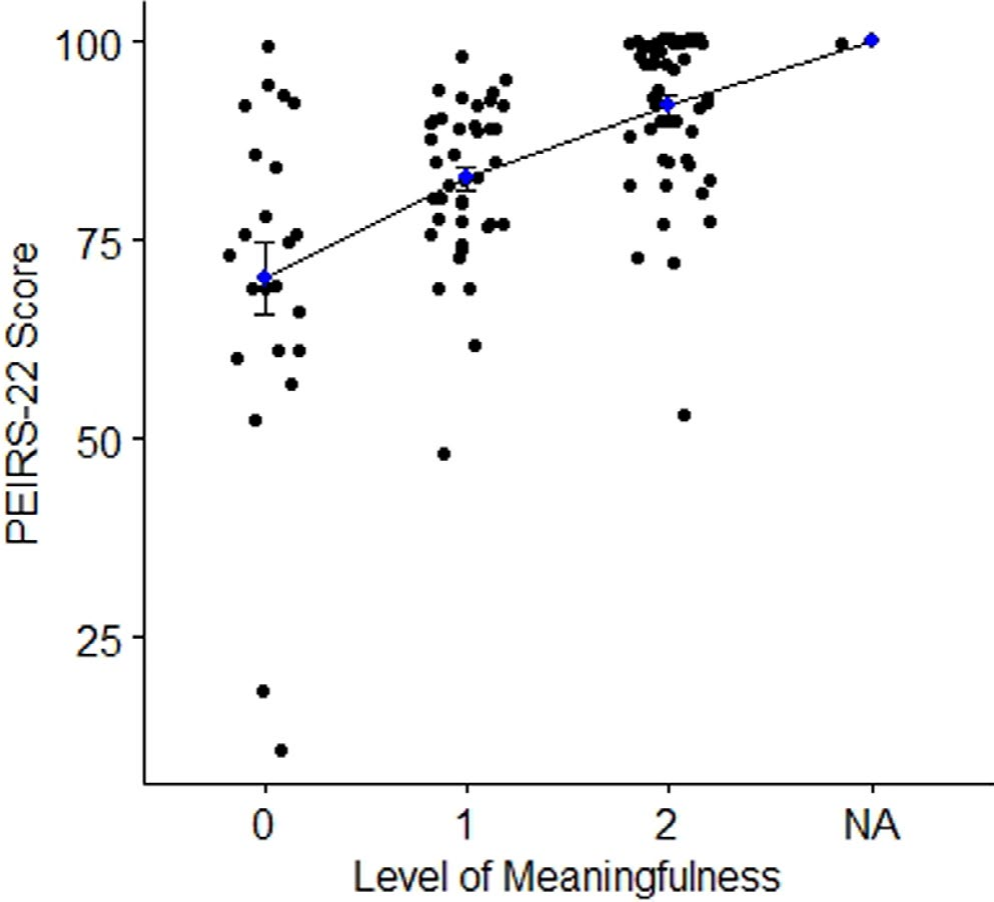
Plot of PEIRS‐22 scores across level of meaningful engagement. The plot shows the mean PEIRS‐22 scores and their standard error bar across three levels of global meaningful patient engagement in research. On the *x*‐axis 0 = not to moderately meaningful, 1 = very meaningful, 2 = extremely meaningful and NA = missing

Correlation between the PEIRS‐22 and global meaningful engagement was 0.61, suggesting the global meaningful engagement measure is appropriate for this analysis. A normality check showed at least three participants had outlying PEIRS‐22 scores, with a statistically significant Shapiro‐Wilk normality test (α < 0.001). The median PEIRS‐22 score was significantly different (Kruskal‐Wallis chi‐squared = 36.42, *df* = 2, *p*‐value = 1.236e‐08) among the groups of global meaningful engagement. Figure 4 illustrates the mean PEIRS‐22 score increased across the three categories of global meaningful engagement. Table 4 shows the comparison between each pair of levels of participants’ PEIRS‐22 scores of global meaningful engagement was statistically significant (α < 0.001). The effect sizes varied as follows: ‘not to moderately meaningful’ with ‘very meaningful’ (*d* = 0.58, small effect size), ‘not to moderately meaningful’ with ‘extremely meaningful’ (*d* = 1.28, moderate effect size) and ‘very meaningful’ with ‘extremely meaningful’ (*d* = 1.04, close‐to‐moderate).

**TABLE 4:** Results of interpretability analysis for PEIRS‐22^a^

Level of global meaningful engagement^b^	PEIRS‐22 score	PEIRS‐22 score
	Median	Mean (standard deviation)
Not to moderately meaningful	73	70.1 (21.9)
Very meaningful	84	82.7 (10.2)
Extremely meaningful	94	92.0 (9.6)

^a^ Comparison between each pair of groups was statistically significant (α < 0.001).

^b^ The numbers in parentheses correspond with the numbers in Figure 3.

## 4 DISCUSSION

In this study, we designed a 22‐item version of the PEIRS (PEIRS‐22) by removing 15 items that either were not consistent with the construct collectively captured by most items or provided little additional information for another item. Psychometric evaluation demonstrated the PEIRS‐22 had good internal consistency, floor and ceiling effect, structural and construct validity, and reliability. Furthermore, this measure has interpretable total scores, and it demonstrated acceptability via the low missing responses. After this study, six patient partner volunteers completed the PEIRS‐22 and found it would likely take 3 to 7 minutes to complete. Finally, calculating the PEIRS‐22 scores is simple, which should attest to a low administrative burden. Overall, our results indicate that the PEIRS‐22 has good measurement properties and could feasibly be completed by patient partners in Canada and the United States.

The results from both the exploratory factor and Rasch analyses indicated the PEIRS‐22 captures a single dominant construct, which we called meaningful engagement in research. The strong factor loading of most items (ie 18 of 22 items are >0.70) and strong corrected item‐total correlations suggest the items fit well with each other and are appropriate for producing summative scores. The PEIRS‐22 scores presented in this study are continuous scores; interval‐level scores could also be generated since the scale may be unidimensional. However, the study sample could benefit from more participants with less favourable scores on the PEIRS‐22 before generating interval‐level scores through a Rasch analysis.

The PEIRS‐22 is comprehensive as it maintains coverage of the eight themes in its conceptual framework. It covers the context, process and outcome/impact of engagement from a patient/family caregiver partner perspective.^49^ When evaluating patient engagement in research, other measures may be needed in addition to the PEIRS‐22 for a broader coverage of the impact of patient engagement.^50^ This broader coverage could include the assessment of acceptability, feasibility, rigour and relevance of research studies as highlighted by the Patient‐Centered Outcomes Research Institute, which may require a range of stakeholders’ views beyond a patient partner perception of what is meaningful.^3^ Furthermore, measuring the impact such as the use of the findings in making health decisions and framing policies might go beyond self‐reported outcomes.^3^

The PEIRS‐22 display of strong internal consistency and good test‐retest reliability results suggests this measure could reliably evaluate meaningful engagement in research for individual patient/family caregiver partners.^39^, ^43^ Furthermore, no statistically significant difference in the median PEIRS scores by demographic characteristics and no item bias suggest the PEIRS‐22 properties may be consistent across those groups.

The PEIRS‐22 produced distinct median scores across the three levels of global meaningful engagement in research. Our results showed that the mean scores can be used as a guide for interpreting the level of meaningful engagement, with a score under 70 meaning a low to moderate level of meaningful engagement, and above 92 meaning extremely high meaningful engagement. Overall, 0 to 100 is the possible range of PEIRS‐22 scores; higher scores are intended to mean higher degree of meaningful engagement. When these benchmark scores are used for comparisons, consider that the smallest detectable change for an individual is about 8% to 13% of the PEIRS‐22’s possible range. This large MDC_90_ is based on SEM which typically varies across a scale. A future study should determine MDC_90_ for different segments of PEIRS‐22’s possible range of scores. Future research could also use known groups and test additional hypotheses to develop benchmarks for interpreting PEIRS‐22 scores in various engagement scenarios.

The higher correlations between PEIRS‐22 scores with each of its own items (≥0.65 even after correlation is corrected for overlap between item and scale) than with the 10 items of the PPEET^29^ suggest the two questionnaires are measuring some different elements of engagement. The moderate correlations (between 0.40 and 0.70) with several of the PPEET items confirm they are measuring similar constructs, but three low correlations (between 0.26 and 0.29) point to divergence of the constructs between the two questionnaires. We speculate this divergence could be partially explained by the focus of PPEET on engagement initiatives by health system organizations, while the PEIRS‐22 focuses on engagement initiatives specific to research. Using the PEIRS‐22 might address the issue of little variability being achieved when using individual PPEET items.^51^

A newer tool, the Patient and Public Involvement Assessment Survey, has been developed and validated to measure satisfaction with patient engagement in basic science and preclinical research.^52^ The PEIRS‐22 covers a broader research scope and may be more appropriate for measuring the quality of patient engagement in research across various patient engagement strategies. As a high‐quality, research‐specific scale, the PEIRS‐22 could be part of a toolkit or curriculum for researchers to monitor their progress in a feedback loop with the people engaging with them. Thus, by using information captured in the PEIRS‐22, researchers and patient partners could improve how they work together.

Our study had limitations. First, the average sample size of 5.3 participants per item for exploratory factor analysis is less than is widely recommended, although it is acceptable.^25^ However, since most factor loadings were large (>0.7) and only one factor was extracted, despite the large number of items, it is likely that the exploratory results are stable.^53^ Second, while a sample size of 100 participants was appropriate for a Rasch analysis, the sample did not include enough people with low degrees of meaningful engagement.^54^ This reduced the potential for stable calibration of items to produce interval‐level scores. A future study sample with more diverse levels of engagement and larger sample size could be achieved via a respondent‐driven sampling approach.^55^ This would be more appropriate for calibrating stable interval‐level scores for the construct manifested from the PEIRS‐22. Third, group numbers precluded analysis between types of partners. Fourth, targeting different groups of participants using different administration modes was a limitation; a future study should investigate whether administration modes impact on the PEIRS‐22 psychometric properties. Finally, the respondents were predominately female or aged between 17 and 65 years. While no studies have provided data on representative demographics of patient and family caregiver partners, our multimodal recruitment that included referrals by researchers and patient partners could have caused recruitment bias. Respondent‐driven sampling with long referral chains could produce a representative sample for this hard‐to‐reach population.^55^

## 5 CONCLUSIONS

The PEIRS‐22, co‐designed with patient partners, is a valid and reliable tool for assessing the degree of meaningful patient/family caregiver engagement in research. It enables standardized assessment of patient/family caregiver engagement in research across a variety of contexts, enables research teams to gather valid information quickly on the quality of patient engagement in research and provides a foundation for comparative effectiveness research on patient/family caregiver engagement strategies.

## 6 DATA SHARING/AVAILABILITY

The data that support the findings of this study are available from the corresponding author upon reasonable request.

## CONFLICT OF INTEREST

The authors have no conflict of interest to declare.

## AUTHOR CONTRIBUTIONS

All authors, except LDH, contributed to the conception and design, or acquisition of data, or analysis and interpretation of data the web survey portion of this study. All authors, including LDH, contributed to the conception, design and acquisition of data of the paper‐based survey portion of the study. CBH draft the initial manuscript, and all authors revised it critically for important intellectual content and approved the version to be published.

## APPENDIX A Items removed from the PEIRS to form the PEIRS‐22

**Table:**

Item	Reason for removal
PR1—I was interested in the issue(s) being researched in the project	Low inter‐item correlations with 21 items between ‐0.22 and 0.19; average = 0.15
PR3—The number of patient partners on the research project team seemed appropriate	Low inter‐item correlations with 10 items between ‐13 and 0.18; average = 0.23
PR4—I understood the objective(s) of the project	Low inter‐item correlations with 11 items between ‐0.09 and 0.19; average = 0.26
PR5—I agreed with the objective(s) of the project	Low inter‐item correlation with 15 items between ‐0.13 and 0.19; average = 0.21
PR6—I understood how I could contribute to the project	Low inter‐item correlations with 15 items between ‐0.13 and 0.19; average = 0.21
PR7—I received sufficient explanation about the project	Low inter‐item correlations with 20 items between ‐0.15 and 0.18; average = 0.15
PR8—I understood my ethical responsibilities for the project	Low inter‐item correlations with 9 items between ‐0.19 and 0.11; average = 0.24
CN2—My preferences for meetings (such as time, duration, location, and format) were considered when planning meetings	Does not fit with Rasch derived construct (fit residual = 3.24). [Rasch Analysis]
CT3—I shared my knowledge within the project team	High residual correlation (>0.4) – too similar to CT1 and CN4 [Rasch Analysis]
T1—Throughout the project, I felt accepted as a member of the research project team	Too high correlations (≥0.78) with T3 and T5
T3—My interactions within the research project team were positive	High residual correlations with other items [Rasch Analysis]
T4—There was mutual respect among the research project team members	Too high correlation (>0.8) with T5
SU3—I was offered sufficient reimbursement for my out‐of‐pocket expenses (such as childcare, parking, and travel) related to the project activities	Low inter‐item correlation (<0.20) with 4 items; average = 0.30
FV2—The research project team was open to receiving my views	Relatively high correlation (≥0.74) with T4 and FV1
BE3—I saw how my contributions could benefit others	Low inter‐item correlation with 10 items between ‐0.11 and 0.18; average = 0.26
